# Engaging Patients in User centered Design for Passive Detection of Dementia and Participation in Brain Health Research in Primary Care

**DOI:** 10.1002/alz70858_102208

**Published:** 2025-12-25

**Authors:** Nicole R Fowler, Nicole R. Fowler, Diana Summanwar, Zina Ben Miled, Phillip M. Drayer, Deanna Willis

**Affiliations:** ^1^ Center for Aging Research at the Regenstrief Institute, Indianapolis, IN, USA; Indiana University School of Medicine, Indianapolis, IN, USA; ^2^ Department of Medicine, Indiana University School of Medicine, IU Center for Aging Research; ^3^ Department of Family Medicine, Indiana University School of Medicine; ^4^ Dept of Electrical and Computer Engineering, Lamar University; ^5^ Department of Family Medicine, Indiana University School of Medicine

## Abstract

Most older adults who have or who are at‐risk of developing dementia rely solely on primary care (PC). PC is the ideal setting to establish guidelines and workflows for detection and diagnostic assessment for dementia, and to facilitate access to pharmacologic and nonpharmacological treatment and research. Indiana University (IU) and IU Health (IUH) are testing using a machine learning algorithm (MLA) to passively detect PC patients who have undetected cognitive impairment or who are at risk of developing dementia in the next 1‐3 years. The feasibility and acceptability of implementing this intervention into clinical use requires engagement from patients and providers to create both the message and the mode by which high risk patients will be engaged in their diagnostic and care journey. The IU/ IUH Brain Health Navigator (BHN) is a PC‐based nurse navigator who conducts evidence‐based assessments for addressable and pathophysiologic causes of cognitive impairment, provides recommendations to the PCP for next steps, answers patient and family questions, and facilitates access to participation in research. This study will test the feasibility and acceptability of using a MLA to (1) detect PC patients who are at high risk of having undetected dementia or developing dementia; (2) engage these high‐risk patients to have a visit with the PC‐based BHN, and (3) increase the number of patients who agree to be contacted for brain health research. To date 20 patients are scheduled to participate in the user‐centered design session and ten diverse clinics have been approached to participate in the pilot.

